# Adipose-derived mesenchymal stromal cells decrease prion-induced glial inflammation in vitro

**DOI:** 10.1038/s41598-022-26628-7

**Published:** 2022-12-29

**Authors:** Arielle J. D. Hay, Tanner J. Murphy, Katriana A. Popichak, Mark D. Zabel, Julie A. Moreno

**Affiliations:** 1 Prion Research Center, College of Veterinary Medicine and Biomedical Sciences, Colorado State University, Fort Collins, CO 80523 USA; 2 Department of Microbiology, Immunology and Pathology, College of Veterinary Medicine and Biomedical Sciences, Colorado State University, Fort Collins, CO 80523 USA; 3 Department of Environmental and Radiological Health Sciences, College of Veterinary Medicine and Biomedical Sciences, Colorado State University, Fort Collins, CO 80523 USA; 4 Present Address: Center for Healthy Aging, Colorado State University, Fort Collins, CO 80523 USA

**Keywords:** Neurodegeneration, Microglia

## Abstract

Prion diseases are characterized by the cellular prion protein, PrP^C^, misfolding and aggregating into the infectious prion protein, PrP^Sc^, which leads to neurodegeneration and death. An early sign of disease is inflammation in the brain and the shift of resting glial cells to reactive astrocytes and activated microglia. Few therapeutics target this stage of disease. Mesenchymal stromal cells produce anti-inflammatory molecules when exposed to inflammatory signals and damaged tissue. Here, we show that adipose-derived mesenchymal stromal cells (AdMSCs) migrate toward prion-infected brain homogenate and produce the anti-inflammatory molecules transforming growth factor β (TGFβ) and tumor necrosis factor-stimulated gene 6 (TSG-6). In an in vitro model of prion exposure of both primary mixed glia and BV2 microglial cell line, co-culturing with AdMSCs led to a significant decrease in inflammatory cytokine mRNA and markers of reactive astrocytes and activated microglia. This protection against in vitro prion-associated inflammatory responses is independent of PrP^Sc^ replication. These data support a role for AdMSCs as a beneficial therapeutic for decreasing the early onset of glial inflammation and reprogramming glial cells to a protective phenotype.

## Introduction

Prion diseases are rare neurodegenerative protein-misfolding diseases (NPMDs) that can be genetic, sporadic, or infectious. This family of diseases result from the native conformation of the cellular prion protein (PrP^C^) misfolding to the infectious form, named PrP-scrapie (PrP^Sc^)^1^. All mammals assessed to date express PrP^C^ in most tissues, with the highest expression found in neural tissue^2^. Once PrP^C^ changes conformation to the beta-sheet rich PrP^Sc^, the misfolded protein has the propensity to form amyloid fibrils and aggregates, which disrupt brain homeostasis^3–5^. Currently, few compounds have been shown to reduce signs of prion disease in mouse models, many of which have toxic effects in the brain or elsewhere^6–9^.

An early sign of prion disease and other NPMDs is neuroinflammation mediated by reactive astrocytes and microglia activation. PrP^Sc^ accumulation causes neuroinflammation and results in oxidative stress, disruption of neural signaling, and glial scarring^10^. The combination of these pathologies leads to synaptic dysfunction, loss of synaptic proteins, and neuronal death^11–14^. As there are limited sources of neurogenesis in the brain, this leads to irreversible neuronal loss and ultimately neurodegeneration. However, mounting evidence supports that PrP^Sc^ itself is not neurotoxic^5,12,13^, and other cellular stress pathways, including glial inflammation, play a major role in disease pathogenesis^12,15,16^. Astrocytes function to promote homeostasis in the brain, including maintenance of the blood brain barrier, uptake of neurotransmitters, and response to infection^17,18^. Astrocyte numbers and inflammatory phenotypes increase in the prion-diseased brain as an early sign of infection, well before clinical signs appear^19–23^. Astrocytes communicate not only with neurons, but also with microglia, the resident macrophages of the brain. Together these cells respond to PrP^Sc^ aggregation by producing proinflammatory cytokines, chemokines, and neurotoxic signals that can contribute to neuronal death. Both astrocytes and microglia can be infected by PrP^Sc^ in vivo and propagate and disseminate infectious prions to neurons^14,24,25^. Interestingly, significant transcriptional changes are seen in glial cells in the prion-infected brain, but few are seen in neurons, further demonstrating the role of glia in disease pathogenesis. Therefore, a critical window exists to treat prion diseases and other NPMDs—the initial inflammation stage induced by reactive glia, prior to the irreversible loss of neurons. We propose harvesting the anti-inflammatory and modulatory functions of mesenchymal stromal cells (MSCs) to target this early stage of disease.

MSCs can be derived from the bone marrow, umbilical cord, and adipose tissue, and can be expanded easily in culture^26^. Adipose-derived mesenchymal stromal cells (AdMSCs) can be taken from patients in a minimally invasive lipectomy^27^. AdMSCs secrete mediators such as the anti-inflammatory cytokines, transforming growth factor β (TGFβ) and tumor necrosis factor-stimulated gene 6 (TSG-6), chemokines, neurotropic factors and growth factors in response to inflammatory conditions^28^. AdMSCs can modulate inflammation in a paracrine manner in a variety of neurological disorders^28,29^, including mouse models of NPMDs such as Alzheimer’s and Parkinson’s diseases^30,31^. AdMSCs target to sites of inflammation and injury through signaling mechanisms involving chemokines, cytokines and growth factors^32^. Moreover, they have been shown to polarize reactive astrocytes and activated microglia toward a neuroprotective phenotype^33,34^.

Significant cross-talk occurs between microglia and astrocytes after insult to the brain. Further elucidating how these cells interact with one another in the context of prion disease is critical to developing therapeutics that alleviate prion-induced neuroinflammation. Microglia show an increased capacity to phagocytose during prion disease and clear PrP^Sc^ at early stages, but this declines as disease progresses^35,36^. In addition, microglia respond to PrP^Sc^ accumulation by secreting tumor necrosis factor alpha (TNFα), interleukin 1 alpha (IL-1*α*) and complement component 1, subcomponent q (C1q), which induce the development of A1 astrocytes, marked by expression of S100β and the complement protein C3^20,23,37^. Unlike resting astrocytes, which promote the formation of neuronal synapses, reactive A1 astrocytes are unable to maintain synapses and secrete unknown neurotoxic signals, resulting in the dysfunction and death of neurons^37^. Reactive astrocytes further activate and induce an M1 phenotype in microglia through the secretion of pro-inflammatory cytokines such as CCL2^38,39^, resulting in increased cytokine production by microglia and migration to sites of inflammation^40^. The complete knock out of TNFα, IL-1α and C1q prevents A1 astrocyte development in prion infected mice, but causes a significant decrease in survival time compared to wild-type (WT) mice^20^. This suggests that A1 astrocytes play a multifaceted role in prion disease through their production of inflammatory cytokines and clearance of infected neurons. Similarly, halting microglial proliferation prior to clinical signs of disease is beneficial in extending survival and decreasing inflammation in prion-infected mice^15^, but ablation of microglia during early-, mid- or late-disease increases astrogliosis and disease progression and decreases survival^23,41^. Total knock-out of microglia decreased survival time in prion-infected mice, despite these mice showing less PrP^Sc^ accumulation. These mice show a unique profile of reactive astrocytes that became activated earlier in disease and demonstrated an increased ability to phagocytose neuronal synapses, suggesting that microglia play a critical role in protecting the prion-infected brain against neurotoxic astrocytes^42^. Together, these findings show that both activated microglia and reactive astrocytes are critical in curtailing disease, but can be detrimental if left unchecked. Although further investigation is necessary to determine the time-point in disease that modulating gliosis would be beneficial, we propose that the ideal treatment for prion-induced inflammation is reducing reactive astrocytes and activated microglia, while preserving their phagocytic and other protective roles.

AdMSCs are a novel therapeutic approach to prion disease, as they adapt in response to the inflammatory milieu in their environment, migrate to regions with high cytokine gradients and can be safely derived and used autologously. Here, we investigate the ability of AdMSCs to decrease prion-induced inflammation and reprogram astrocytes and microglia to a protective phenotype.

## Results

### Characterization of adipose-derived mesenchymal stromal cells isolated from mouse visceral fat and expanded in culture

**Figure 1:**
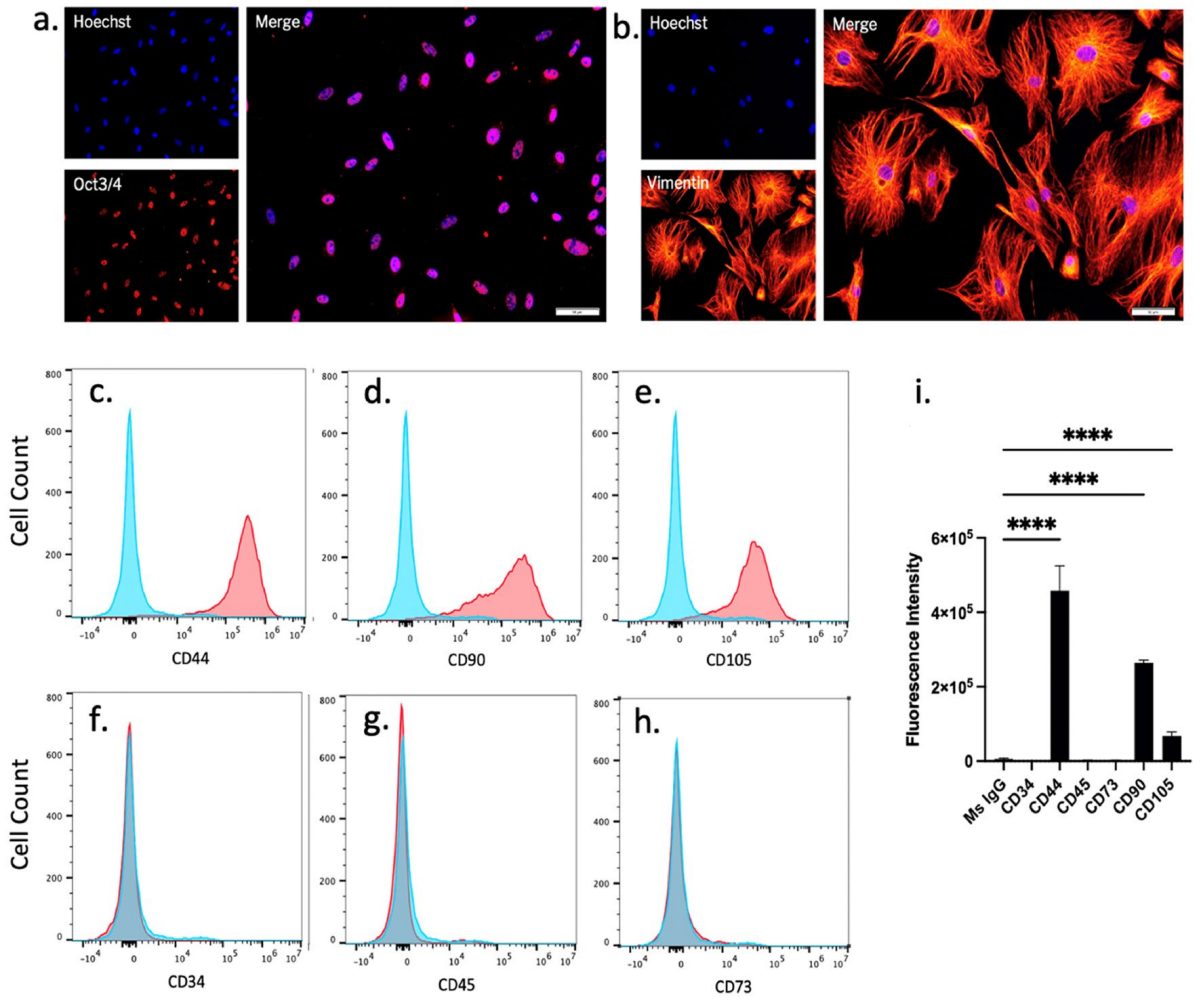
Characterization of adipose-derived mesenchymal stromal cells isolated from mouse visceral fat and expanded in culture. AdMSCs express the (**a**) undifferentiated cell marker Oct3/4 and (**b**) the structural protein Vimentin. AdMSCs express (**c**) CD44, (**d**) CD90 and (**e**) CD105 but not (**f**) CD34, (**g**) CD45 or (**h**) CD73 (red histograms) compared to IgG control (blue histograms). (**i**) Fluorescent intensities of AdMSC profile quantified in FlowJo. Scale bar = 50 µM. Representative histograms from three biological replicates with 10,000 events each. Two-way ANOVA and post-hoc Tukey test, error bars = SEM, ****p < 0.0001.

Adipose-derived mesenchymal stromal cells (AdMSCs) were isolated from the abdominal adipose tissue of adult C57Bl/6 mice and expanded to passage 3. Characteristic AdMSC markers were analyzed through immunofluorescence and flow cytometry. Note that expression of markers was not uniform across all cells, suggesting that this is a heterologous population. AdMSCs express the undifferentiated cell marker Oct3/4 (Fig. 1a) and the structural marker Vimentin (Fig. 1b), as shown by immunofluorescence. These cells express membrane markers CD44 (97.1%), CD90 (74.2%) and stem cell marker CD105 (68.9%) (Fig. 1c-e) and do not express hematopoietic stem cell markers CD34 (> 0.1%) or CD45 (3.0%) (Fig. 1f-g), nor do they express significant CD73 (2.1%) (Fig. 1h), as shown by quantification of flow cytometry fluorescence intensity (Fig. 1i).

### AdMSCs produce anti-inflammatory molecules in response to inflammatory cytokines and prion-infected brain homogenate

It is well established that AdMSCs produce anti-inflammatory molecules in response to inflammatory cytokines^43,44^. To stimulate the upregulation of anti-inflammatory genes, AdMSCs were treated with 10 ng/ml TNFα or 200 ng/ml IFNγ^43^. To determine if AdMSCs upregulate genes when exposed to the cytokine milieu of the prion-infected brain, AdMSCs were exposed to 22L-infected or normal brain homogenates (NBH). Cytokines or brain homogenates were diluted in media and incubated with AdMSCs for 1, 4, 8, 12 or 24 h before RNA was collected. Untreated samples were incubated with media only.

**Figure 2:**
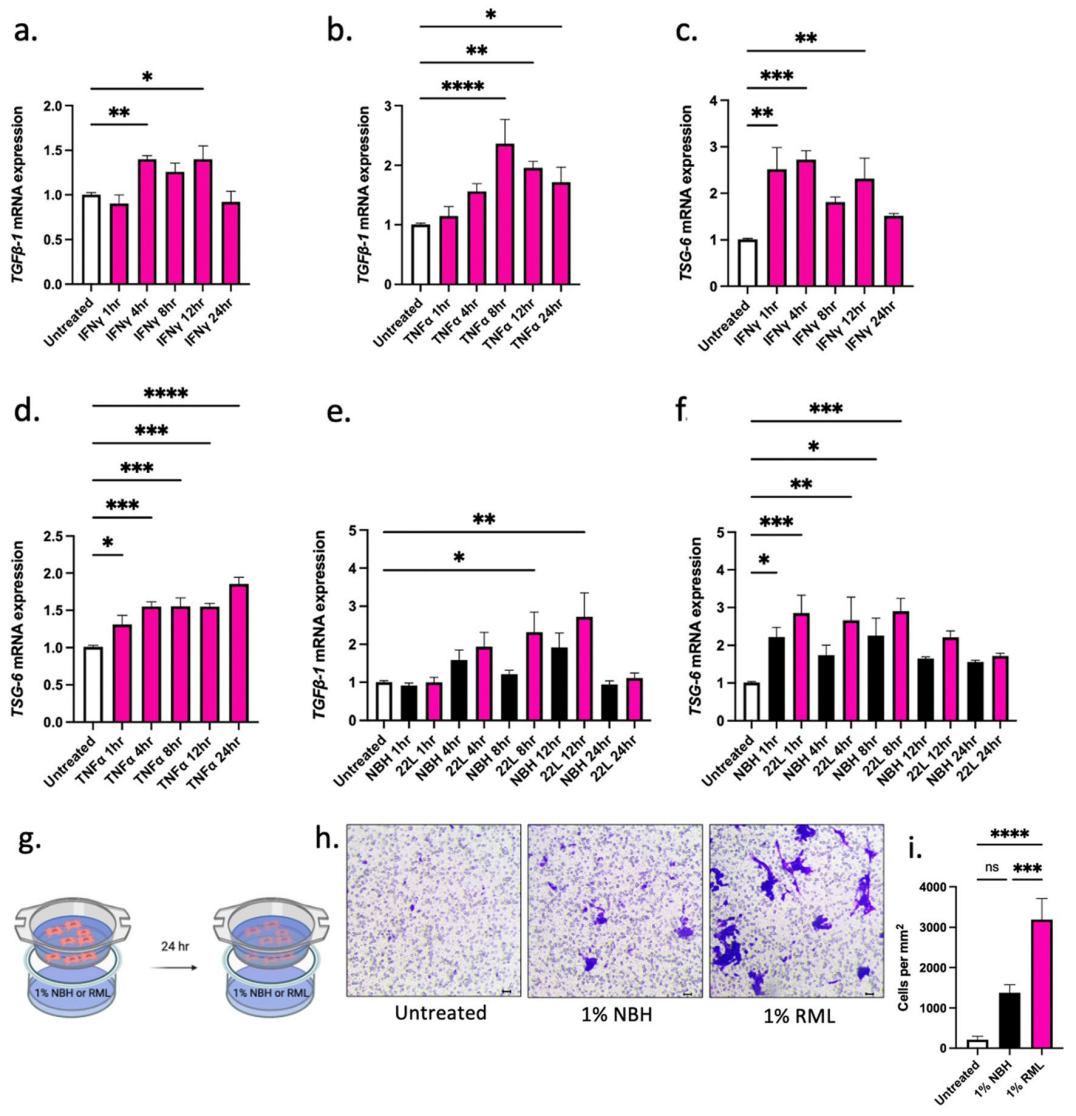
AdMSCs produce anti-inflammatory molecules in response to inflammatory cytokines and prion-infected brain homogenate. Exposing AdMSCs to IFN-γ or TNFα increases (**a**, **b**) *TGFβ-1* and (**c**, **d**) *TSG-6* mRNA. Exposing AdMSCs to 22L prion-infected but not normal brain homogenate (NBH) increases (**e**) *TGFβ-1*. Both NBH and 22L brain homogenate increase (**f**) *TSG-6* mRNA. Two biological replicates, each with three technical replicates, all analyses normalized to *β-actin*. (**g-i**) TNFα stimulated AdMSCs migrate toward RML prion brain homogenate after 24-h exposure. Cell counts were taken from four randomly selected fields of view at 10 × magnification. Scale bar = 10 µm. Three biological replicates each with three technical replicates. One-way ANOVA and post-hoc Tukey test, error bars = SEM, *p < 0.05, **p < 0.01, ***p < 0.001, ****p < 0.0001, ns = not significant. Graphic created with BioRender.com.

AdMSCs increase expression of the anti-inflammatory cytokine *TGFβ-1* mRNA when exposed to the cytokines IFNγ and TNFα. Treatment with IFNγ triggered the production of *TGFβ-1* mRNA after 4 and 12 h (*p* < 0.01; *p* < 0.05) (Fig. 2a). Treatment with TNFα produced a maximum increase in *TGFβ-1* mRNA after 8 h (*p* < 0.001) and *TGFβ-1* mRNA was sustained at 12 and 24 h (*p* < 0.01; *p* < 0.05) (Fig. 2b). Additionally, mRNA for tumor necrosis factor-stimulated gene 6 (TSG-6) was produced by AdMSCs when exposed to IFNγ and TNFα. Treatment with IFNγ triggered the production of *TSG-6* mRNA after 1, 4 and 12 h (*p* < 0.01; *p* < 0.001; *p* < 0.01) (Fig. 2c). A dramatic increase in *TSG-6* mRNA was seen after treating AdMSCs with TNFα, which resulted in an increase after 1 h (*p* < 0.05), increased further at 4, 8 and 12 h (*p* < 0.001), and was sustained until 24 h (*p* < 0.0001) (Fig. 2d).

Treatment with brain homogenates from 22L-infected mice was sufficient to stimulate *TGFβ-1* mRNA production in AdMSCs after 8 h (*p* < 0.05). This increase was maintained at 12 h (*p* < 0.01), but at 24 h had returned to baseline (Fig. 2e). *TSG-6* mRNA showed a slight increase in cells treated with normal brain homogenates (NBH) at 1 and 8 h (*p* < 0.05). A greater increase was seen in *TSG-6* mRNA after treatment with 22L brain homogenates after 1 h (*p* < 0.001), and this was sustained after 4 h (*p* < 0.01) and 8 h (*p* < 0.001) before returning to baseline at 12 h (Fig. 2f).

Pre-treating MSCs with TNFα can improve their ability to migrate to sites of inflammation^45,46^. The ability of AdMSCs to migrate to sites of prion-induced inflammation was assessed in vitro using a cell migration assay. AdMSCs were stimulated for 24 h with TNFα, serum starved for 4 h, then plated in inserts above media containing vehicle-only or 1% normal or RML-scrapie brain homogenate (Fig. 2g). Cells were incubated for 24 h and the cells on the upper side of the insert were removed. Cells on the bottom of the insert were stained and four randomly selected fields of view were imaged and counted at 10 × magnification (Fig. 2h). No statistical difference was seen in cell counts between cells exposed to vehicle (mean = 7.267) and NBH (mean = 65.18). Significantly more cells migrated toward RML brain homogenate (mean = 146.6) compared to both vehicle (*p* < 0.0001) and NBH (*p* < 0.001) (Fig. 2i).

### Stimulated AdMSCs decrease inflammatory phenotypes in BV2 microglia

**Figure 3:**
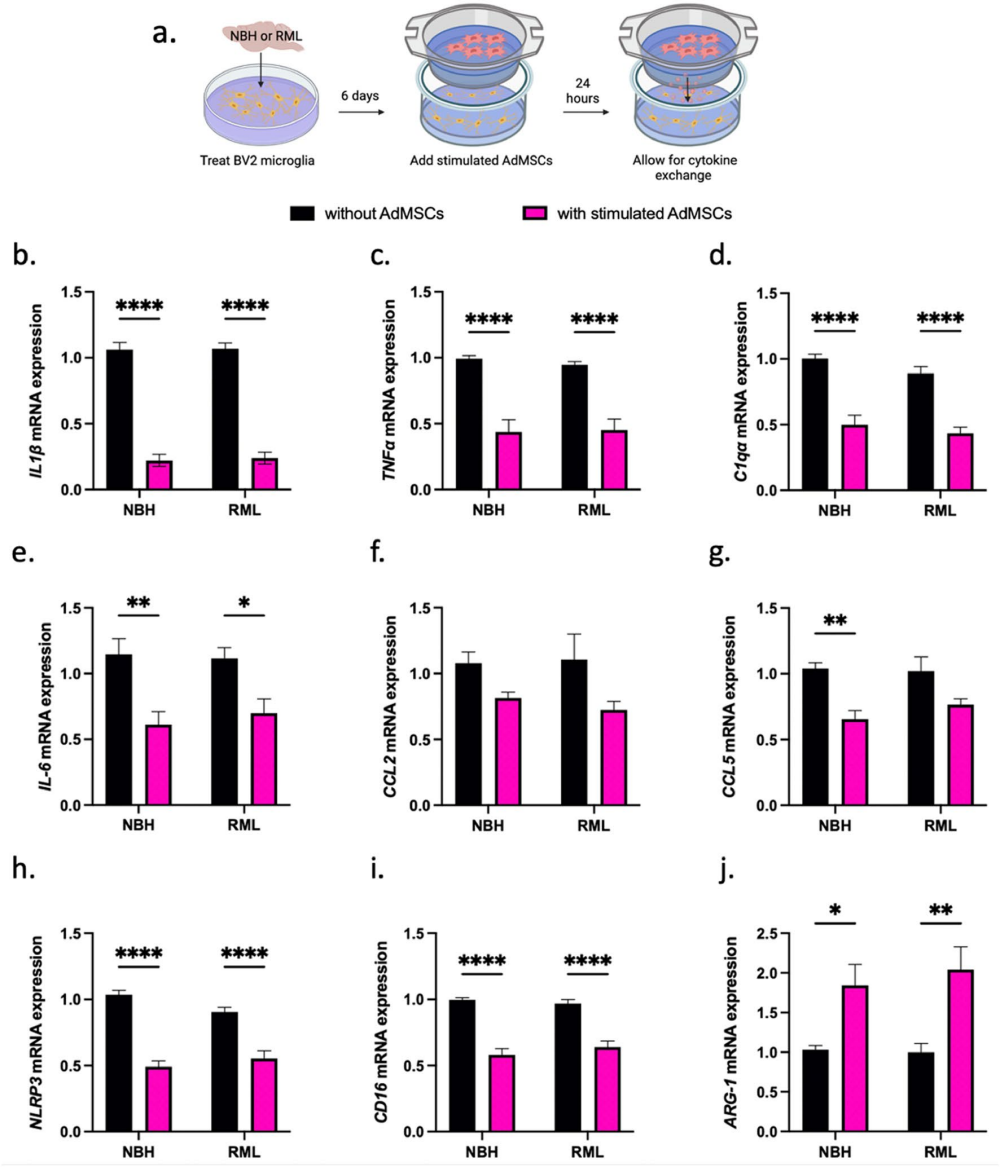
Stimulated AdMSCs decrease inflammatory phenotypes in BV2 microglia. (**a**) BV2 microglia were exposed to RML prion brain homogenate or normal brain homogenate (NBH) for 6 days, then co-cultured for 24 h with TNFα-stimulated AdMSCs. This resulted in a decrease in inflammatory cytokines (**b**) IL1β, (**c**) TNFα, (**d**) C1qα, and (**e**) IL-6. No significant change was seen in (**f**) CCL2 and a decrease was seen only in the NBH treatment for (**g**) CCL5. A decrease was seen in mRNA for the (**h**) NLRP3 inflammasome and for M1 marker (**i**) CD16. An increase was seen for the M2 marker (**j**) Arg-1. Three biological replicates, with three technical replicates, all analyses normalized to *β-actin*. Two-way ANOVA and post-hoc Tukey test, error bars = SEM, *p < 0.05, **p < 0.01, ***p < 0.001, ****p < 0.0001. Graphic created with BioRender.com.

To determine whether AdMSCs influence microglia-derived inflammatory molecules and markers of polarized glia, the BV2 murine microglia cell line was utilized. BV2 cells were exposed to 0.1% RML or NBH for 3 days. Stimulating AdMSCs with TNFα causes an increase in production of the anti-inflammatory genes *TGFβ-1* and *TSG-6* (Fig. 2b, d). Therefore, we pre-treated AdMSCs with 10 ng/ml TNFα for 24 h prior to co-culturing with BV2 cells. At 6 days post-prion exposure, BV2 cells were co-cultured with AdMSCs for 24 h before BV2 RNA was isolated (Fig. 3a). Note that after stimulation and washing with PBS, TNFα is not significantly detectable by ELISA in AdMSC media (Supplemental Fig. 1), demonstrating that residual TNFα in the media is not directly stimulating BV2 cells.

No differences were seen in transcript levels for inflammatory markers between NBH and RML-treated BV2 cells at the timepoint used in this study. However, AdMSCs drastically decreased transcription of pro-inflammatory markers in BV2 cells, regardless of exposure to RML or NBH. A significant decrease was seen in *IL1β* for both NBH-treated and RML-treated BV2 cells (*p* < 0.0001) (Fig. 3b). Intriguingly, mRNA for the genes *TNFα* and *C1qa* were decreased in both NBH-treated and RML-treated BV2 cells (*p* < 0.0001) (Fig. 3c, d). These genes are critical for microglia-derived molecules that polarize astrocytes to the A1 reactive phenotype^37^. A significant decrease was seen in *IL-6* mRNA for both NBH-treated (*p* < 0.01) and RML- treated BV2 cells (*p* < 0.05) (Fig. 3e). No significant decrease was seen in mRNA for the inflammatory cytokine *CCL2* in NBH or RML-treated BV2 cells co-cultured with AdMSCs (Fig. 3f). A decrease was seen in *CCL5* mRNA, but was only significant in the NBH-treated glia (*p* < 0.01) (Fig. 3g). Co-culturing with AdMSCs decreased mRNA for the NLRP3 inflammasome (*p* < 0.0001) (Fig. 3h) and the M1 microglial gene *CD-16* in both NBH-treated and RML-treated BV2 cells (*p* < 0.0001) (Fig. 3i). mRNA for *Arg-1*, a marker for M2 microglia, was increased for both NBH-treated (*p* < 0.05) and RML-treated BV2 cells (*p* < 0.01) (Fig. 3j). AdMSCs that were not stimulated with TNFα were sufficient to change expression of some genes in brain homogenate-treated BV2 cells, but did not show as robust of an effect compared to pre-treated/stimulated AdMSCs (Supplemental Fig. 2).

### Co-culture with AdMSCs decreases mRNA for inflammatory genes in infected glia

**Figure 4:**
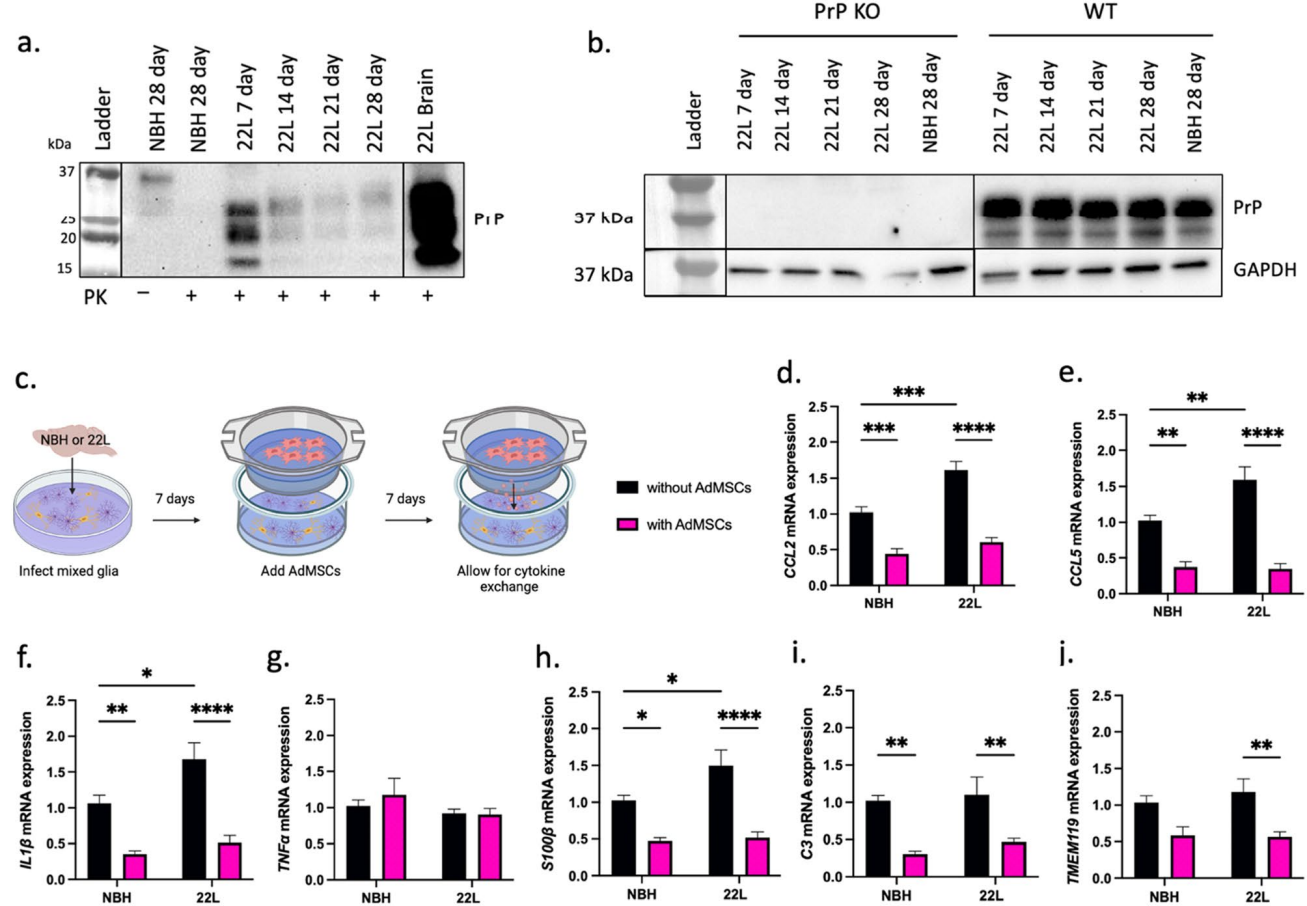
Co-culture with AdMSCs decreases mRNA for inflammatory genes in infected glia. (**a**) Wild-type mixed glia can be infected by being exposed to 0.1% 22L brain homogenates (diluted in media) and maintain infection. PK treatment of glia to remove PrP^C^ signal demonstrates that PrP^Sc^ remains in 22L-treated, but not NBH treated glia. An example of a terminal 22L brain (20% brain homogenate in PBS) used to infect these cells is in right-most lane (full exposure blot in Supplemental Data Fig. 4). (**b**) Total PrP signal (no PK digestion) demonstrates that PrP knock-out glia cannot be infected with 22L prions and maintain infection, indicating that signal or changes in gene expression is not due to residual brain homogenate on cells. Infected wild-type glia show continuous expression of PrP^C^. Blots are cropped and stitched together to remove empty lanes. (**c**) Mixed glial cultures were exposed to normal (NBH) or 22L-prion infected brain homogenates and incubated for 7 days, then co-cultured with or without AdMSCs for an additional 7 days. A decrease was seen in mRNA for inflammatory markers (**d**) CCL2, (**e**) CCL5, and (**f**) IL1β, but not (**g**) TNFα. Reactive astrocyte markers (**h**) S100β and (**i**) C3, and the microglia-specific marker (**j**) TMEM119 also decreased. Four biological replicates, with three technical replicates, all analyses normalized to *GAPDH*. Two-way ANOVA and post-hoc Tukey test, error bars = SEM, *p < 0.05, **p < 0.01, ***p < 0.001, ****p < 0.0001. Graphic created with BioRender.com. Full images of blots are available in Supplemental Figures.

Although microglia play a unique and critical role in the prion infected brain^47^, elucidating the effects AdMSCs have on both astrocytes and microglia, and the cross-talk between these cell types, is essential. To best model the prion-infected brain in vitro, primary mixed glial cultures were utilized. These cultures are derived from cortices of C57Bl/6 mice at zero to two days old and contain both astrocytes and microglia^38^. Confirmational western blot analysis shows that exposure of glial cultures to 22L brain homogenate for 3 days is sufficient for infection, as PrP^Sc^ accumulation is detectable at 7-, 14-, 21- and 28-days dpi (Fig. 4a). It is well established that host expression of PrP is critical for infection^48^. To determine that PrP signal was not due to residual brain homogenate on mixed glial cultures, PrP knock-out glia were treated with 22L brain homogenate for 7 to 28 days. No residual PrP was detected in these cells, while PrP remained consistent in infected WT glia throughout this time-course (Fig. 4b). Together, these data show that glia can be infected and maintain infection for at least 28 days, and that this is newly synthesized PrP^Sc^. Glia at passage 1 were plated at 100,000 cells per well. 24 h later, glial cultures were infected with media containing 0.1% 22L or NBH. At 3 dpi, cells were washed twice with PBS and new media was added. At 7 dpi, a co-culture system was established by adding AdMSCs to inserts. Glia were co-cultured with AdMSCs for 7 days (Fig. 4c) before RNA was isolated from the glia.

A significant increase in *CCL2*, *CCL5, IL1β* and *S100β* mRNA was seen in glial cells infected with 22L compared to NBH (*p* < 0.001; *p* < 0.01; *p* < 0.05; *p* < 0.05, respectively). After co-culturing for 7 days with AdMSCs, a decrease in *CCL2* mRNA was seen in both NBH-treated (*p* < 0.001) and 22L-infected cells (*p* < 0.0001) (Fig. 4d). *CCL5* mRNA decreased in NBH-treated and 22L-infected glia co-cultured with AdMSCs (*p* < 0.01; *p* < 0.0001) (Fig. 4e). Likewise, co-culturing with AdMSCs decreased *IL1β* mRNA in NBH-treated and 22L-infected glia (*p* < 0.01; *p* < 0.0001) (Fig. 4f). No significant changes were seen in *TNFα* mRNA expression (Fig. 4g).

Both overall astrocyte number and the number of C3 + reactive astrocytes increase in the prion-infected brain^20,49^. Here, we show that co-culturing with AdMSCs decreased both the pan-astrocyte marker *S100β* in NBH and 22L-treated glia (*p* < 0.5; *p* < 0.0001) (Fig. 4h) and C3 in NBH and 22L-treated glia (*p* < 0.01; *p* < 0.01) (Fig. 4i). The number of microglia is also known to increase in prion infection^15^, although analysis of TMEM119, a marker for a subset of microglia,^50^ did not recapitulate this in our cell model. Co-culturing with AdMSCs did however decrease the amount of TMEM119 mRNA in 22L-infected glia (*p* < 0.01) (Fig. 4j).

### Protection against glial inflammation is independent of PrP^Sc^

**Figure 5:**
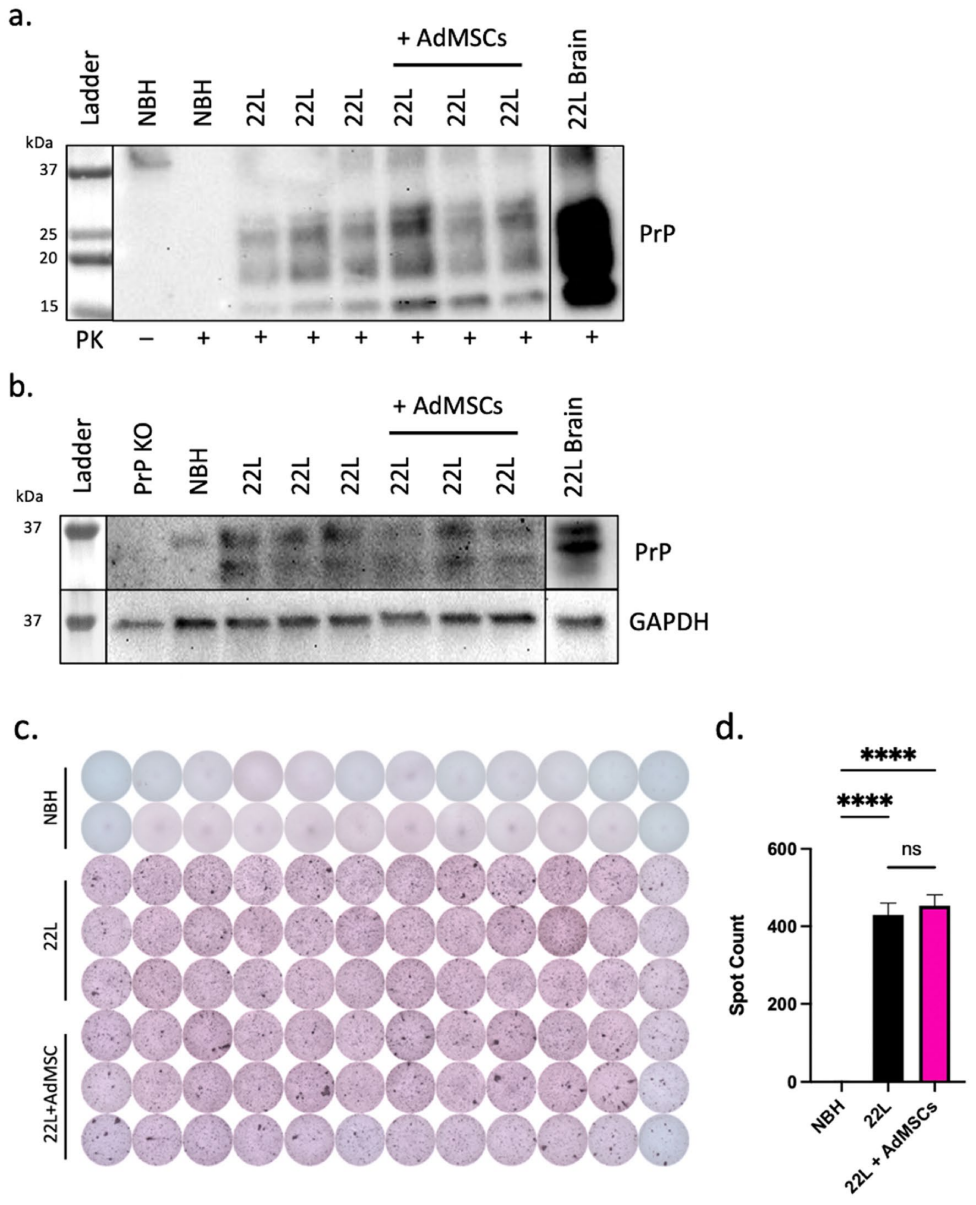
Protection against glial inflammation is independent of PrP^Sc^. Infected mixed glial cells were cocultured with AdMSCs and western blot was used to analyze (**a**) PrP^Sc^ and (**b**) PrP^C^. 22L-infected brain homogenate positive control (lower exposure) in right-most lanes. (**c**) Scrapie cell assay was used to analyze PrP^Sc^ spot counts. Representative image from three separate experiments. (**d**) Spot counts analyzed with One-way ANOVA and post-hoc Tukey test, error bars = SEM, ****p < 0.0001, ns = not significant.

Co-culture systems were set up as described above. After 7 days of infection and an additional 7 days of co-culturing with AdMSCs, cell lysates were taken from glial cells and analyzed for PrP^Sc^ expression using western blot. No significant differences were seen between amount of PrP^Sc^ from infected cells co-cultured with AdMSCs to those without (Fig. 5a). Samples were analyzed for PrP^C^ with similar findings (Fig. 5b). No changes in PrP^Sc^ spot counts were seen by scrapie cell assay (Fig. 5c and d). This suggests that although AdMSCs have a significant impact on the cytokine expression of glial cells, they do not have any direct or indirect effects on the accumulation of PrP^Sc^ in these glial cells.

## Discussion

The prion-infected brain upregulates multiple inflammatory cytokines and chemokines associated with signaling pathways such as pro-inflammatory transcription factor, Nuclear Factor-kappa B (NF-κB), including IL1β, TNFα, and CCL2 and CCL5^51^. Significant changes in gene expression and glial cell activation begin as early as 70 days post infection (dpi) in infected mice^13,52^. At 70 dpi, there is a shift in the number and profile of astrocytes, demonstrated by an increase in GFAP + and S100β + cells, as well as markers specific to the reactive A1 phenotype, namely C3^20,37^. The classic A1 astrocyte phenotype is induced by activated microglia secreting TNFα, C1qa, IL1α and IL1β^37^. This activation initiates a positive feedback loop, as reactive astrocytes produce chemokines such as CCL2, which induces the M1 phenotype in microglia^39^. Contrary to the homeostatic, neuroprotective role that A2 astrocytes and M2 microglia play in the brain, A1 astrocytes have neurotoxic effects^37^ and M1 microglia produce inflammatory cytokines such as TNFα, IL1β and nitric oxide^39^. The removal of A1 astrocytes leads to a decrease in microglial activation, but also accelerates disease, suggesting that these cells play a critical role in host protection^20^.

It should be noted that A1 astrocytes are not the only phenotype involved in prion infection. At terminal stages, prion-infected mice have a unique profile of astrocytes, including those with both A1 and A2 markers^20,23^. In mice lacking microglia altogether, a subset of reactive astrocytes was found, characterized by the expression of C3, LCN2 and GFAP. This neurotoxic phenotype likely contributed to the mice succumbing to disease more rapidly^42^. Various studies have shown that prevention of microglial proliferation prior to clinical signs of disease lengthens survival in prion-infected mice, but ablation of microglia proves to be detrimental^15,41,42^. Therefore, to properly treat early inflammation in prion disease, a fine balance must be established to decrease the number of activated glia without eliminating them altogether. Further studies are required to understand the unique phenotypes of astrocytes involved in prion infection, and the neurotoxic signals they secrete. A1 astrocytes retain a neurotoxic phenotype in vitro, even after the removal of inflammatory cytokines. However, treatment with TGFβ-1 or fibroblast growth factor (FGF) are sufficient to revert A1 astrocytes back to a neuroprotective A2 phenotype^37^. Thus, not only is it critical to decrease soluble factors which contribute to glial activation and neuronal death, but also introduce anti-inflammatory factors that can reprogram glia to a protective phenotype.

Here, we demonstrate that adipose-derived mesenchymal stromal cells (AdMSCs), isolated from the peritoneal visceral fat of mice and expanded in culture (Fig. 1), respond to inflammatory cytokines and prion-infected brain homogenate by increasing production of the anti-inflammatory genes *TGFβ-1* and *TSG-6* (Fig. 2), both of which decrease reactive astrogliosis^34,37^. Moreover, MSC-derived TSG-6 inhibits LPS-induced M1 polarization of microglia and promotes M2 polarization^33^.

To elucidate the role AdMSCs have on the phenotype of BV2 microglia and primary mixed glia that had been exposed to prions, we set up co-culture assays. Notably, we did not see any differences between NBH- and RML-treated BV2 cells at 7 days post exposure, suggesting that the inflammation was not prion associated. However, adMSCs were able to reduce transcripts of inflammatory cytokines induced by brain homogenate in general. Previous studies using BV2 cells treated with prions have looked at transcript levels after 18 h and used purified PrP^Sc^^53^, whereas we selected to use brain homogenate to better mimic the conditions of the prion-infected brain. Our longer timepoints for both BV2 and mixed glial cultures were chosen to ensure that both glia and AdMSCs were not responding to inflammatory signals within the brain homogenates.

Consistent with other studies, we show that cytokine-stimulated MSCs increase production of small anti-inflammatory molecules^43,44^, TGFβ-1 and TSG-6, with the highest expression between 8 and 24 h (Fig. 2a-d). Therefore, we stimulated AdMSCs with TNFα 24 h prior to co-culture with BV2 cells (Fig. 3a) to maximize production of inflammatory modulators. MSCs change phenotype of microglia from the activated and inflammatory M1 phenotype to the neuroprotective M2 phenotype^33,54,55^. Microglia in the prion-infected brain have increased M1 markers^23,25,39^, although the role of M1 and M2 microglia has only begun to be elucidated in prion pathogenesis^23,47,56^.

Here, we show that AdMSCs dampen prion-induced glial inflammation in vitro, supporting the role of NF-κB signaling as an early indicator of prion infection^52,53,57^. In our co-culture systems, a number of NF-κB-mediated secreted cytokines and chemokines (IL1β, CCL2 and CCL5) decrease in prion infected mixed glial cells (Fig. 4d-f). In BV2 microglia that were exposed to normal or prion-infected brain homogenates and co-cultured with AdMSCs, the NF-κB-associated genes IL1β, TNFα, and IL-6 decrease (Fig. 3b, c and e). Previous studies demonstrate that inhibition of NF-κB signaling in prion disease is neuroprotective^57,58^, suggesting a potential mechanism that AdMSCs may use to decrease inflammation. Because these molecules are also implemented in other signaling pathways, this study alone is not sufficient in determining that AdMSCs have an effect on prion-induced NF-κB signaling. However, it is recognized that mesenchymal stromal cells decrease NF-κB in astrocytes activated by LPS exposure, specifically through the production of TSG-6^34^. Further studies are required to determine if AdMSCs have similar effects in prion models.

A second pathway implicated in in vitro models of prion disease is the Nod-Like Receptor Protein 3 (NLRP3) inflammasome, reported as the primary source of microglia-derived IL1β in prion infection^59^. Although the role of the NLRP3 inflammasome in prion mouse models remains controversial^60^, it is a known marker of prion-induced inflammation in both primary microglia and BV2 cells^59,61^. Co-culturing prion-infected primary mixed glia and prion-exposed BV2 cells with AdMSCs yields a significant decrease in IL1β, a key cytokine produced by—but not limited to—the NLRP3 inflammasome pathway. Further evidence that AdMSCs may mechanistically influence this pathway is measured by the significant decrease in mRNA for *NLRP3* in BV2 cells (Fig. 3h).

In our infected primary cell cultures containing both astrocytes and microglia, we show dramatic reduction in mRNA from the inflammatory cytokines CCL2, CCL5 and IL-1β, but not TNFα (Fig. 4d-g), when co-cultured with AdMSCs. Additionally, co-culturing with AdMSCs affected astrocyte phenotype, contributing to a decrease in the A1 astrocyte marker C3 and the reactive astrocyte marker S100β (Fig. 4h-k), which are associated with prion and other neurodegenerative diseases^20,37,50^. mRNA for the microglia-specific marker TMEM119 was also decreased (Fig. 4j), suggesting that fewer TMEM119 + microglia were present in the 22L-infected cultures that were treated with AdMSCs. Together, these data suggest that AdMSCs secrete factors that induce polarization of astrocytes and microglia toward an anti-inflammatory and neuroprotective state and reduce their production of inflammatory signals.

A limitation of this study is the time frame at which markers of astrogliosis and inflammation are upregulated in our mixed glia model. We see a significant increase in IL1β, CCL2, CCL5 and S100β at 14 dpi in 22L-infected mixed glial cultures. However, at this same time point we do not see significant changes in TNFα, C3, or TMEM119. We hypothesize that this is due to the transient nature of many markers in the brain during prion disease, which show changes at various timepoints in disease^23,52^, and these genes remained unchanged at the timepoint we analyzed in vitro. It is well established that markers such as C3 and TNFα are increased in the prion-infected brain^20,23^, but this may not be recapitulated in a cell culture model at 14dpi. TMEM119 is specific to microglia and has been demonstrated to mark a unique subset. TMEM119 transcript levels, but not protein levels, are shown to increase in Alzheimer’s disease^62^, and TMEM119 + cell counts decrease in the brains of terminal prion-infected mice, although this appears to be region specific^47^. Further investigation may be required to understand the role of this protein in neurodegenerative disease, and the effects AdMSCs may have on its expression.

Co-culturing stimulated AdMSCs with brain homogenate exposed BV2 microglia for 24 h decreased IL-6 and TNFα, but not CCL2 and CCL5 (Fig. 3c-g). Conversely, mixed glial cultures containing astrocytes demonstrated less CCL2 and CCL5 mRNA (Fig. 4d-e) when co-cultured with AdMSCs, but showed no changes in IL-6 (data not shown) or TNFα (Fig. 4g). This suggests that microglia and astrocytes are differentially affected by AdMSCs. There is significant cross-talk between microglia and astrocytes to induce the unique glial profile seen in NPMDs such as prion disease. Some is well characterized, such as microglia-derived TNFα inducing an A1 phenotype in astrocytes^20,37^, and astrocyte-derived CCL2 to polarizing microglia to an M1 state^38,39^. However, much of this cell-to-cell communication is poorly understood, and studies that reduce or remove specific cell types, such as A1 astrocytes or microglia, have conflicting results^15,20,41,42,47^.

MSCs have the capacity to not only decrease inflammatory signaling, but to decrease protein aggregates. AdMSCs and AdMSC-derived exosomes have membrane-bound nephrilysin, an enzyme that degrades protein aggregates such as amyloid-β. A decrease in amyloid-β in both cell^63^ and mouse models of Alzheimer’s disease occurs after tail injection of MSC cells or exosomes^64,65^. Although prion disease shares similarities with Alzheimer’s disease, the prion-infected brain contains a unique subset of glial cells^23,25,57^, suggesting that treatments against Alzheimer’s may not have the same effects against prion disease. To determine if similar effects could be seen in prion disease, co-culture systems of infected glia and AdMSCs were analyzed for abundance of PrP^Sc^ using both western blot and scrapie cell assay. AdMSCs did not show an ability to decrease PrP^Sc^ in the context of persistent infection in glial cells, suggesting that the protective capacity of AdMSCs in prion infection is independent of PrP^Sc^. This is consistent with previous studies, which failed to report changes in PrP^Sc^ deposition after therapeutic intervention of gliosis in prion-infected mice, despite observations of increased survival and improved clinical and behavioral signs^15,54,58^.

The therapeutic advantages of mesenchymal stromal cells (MSCs) are promising, particularly in their ability to regulate inflammation by secreting anti-inflammatory small molecules and promote angiogenesis and neurogenesis^28,29^. The secretome of these cells includes anti-inflammatory cytokines and chemokines, growth factors, microRNA and messenger RNAs^66^. Intriguingly, these cells can be taken autologously from patients via a relatively non-invasive procedure^27^, suggesting a realistic approach to individualized treatment. The capacity of MSCs to modulate inflammation in a paracrine manner is shown in a variety of neurological disorders^28^, including NPMDs such as Alzheimer’s and Parkinson’s^30,31,67^. Here, we have shown that MSCs derived from adipose tissue are effective in decreasing inflammation in an in vitro model of prion-induced gliosis. Our data suggest that AdMSCs can decrease glial inflammation consistent with a shift from a neuroinflammatory phenotype toward a phagocytic, neuroprotective role. These data support AdMSCs as a promising therapy to decrease glial inflammation, an early indicator and key characteristic associated with neuronal cell death, but not necessarily in clearance of PrP^Sc^, which necessitates further interrogation. However, taken together with these data, AdMSCs hold exciting value in combatting glial inflammation associated with neurodegenerative disease, especially if combined with other potential therapies designed to decrease PrP^Sc^ in the brain. Future work is underway to confirm the potential of AdMSCs to reprogram the glial response to in vitro and in vivo prion infection and perhaps other NPMDs.

## Materials and methods

### Animal care and ethics statement

Mice were euthanized by deeply anaesthetizing with isoflurane followed by decapitation. All mice were bred and maintained at Lab Animal Resources, accredited by the Association for Assessment and Accreditation of Lab Animal Care International, in accordance with protocols approved by the Institutional Animal Care and Use Committee at Colorado State University. The Institutional Biosafety committee at CSU along with lab safety protocols were followed for BSl2 facility and procedures. All animal experiments followed the ARRIVE 2.0 guidelines, including study design, animal numbers, randomization and statistical methods.

### Brain homogenates

C57Bl/6 (Jackson Laboratory) mice were intracranially inoculated with 30µl of 1% 22L or Rocky Mountain Laboratories (RML) strains of mouse-adapted prions, or normal brain homogenate (NBH). Mice were monitored for weight loss and clinical signs of prion disease and euthanized after showing signs of terminal illness. 20% brain homogenates in phosphate-buffered saline (PBS) were made using beads and a tissue homogenizer (Benchmark Bead Blaster 24) and stored at -80C. Brain homogenates were aliquoted and treated with UV light for 30 min to sterilize before being used for cell culture.

### Isolating and maintaining AdMSCs

Female and male adult C57Bl/6 mice were euthanized and the abdominal adipose tissue was dissected, placed in Hank’s Buffered Saline Solution containing 25% Trypsin (HyClone, 0.25%) and cut into small chunks. Adipose tissue was dissociated by incubating with a mixture of 200U/ml DNase-I (Roche) and 400U/ml Stemxyme (Worthington Biochemical Corporation) in DMEM/F12 media (Caisson Labs) at 37C for 1 h. The tissues were centrifuged at 4C for 5 min at 1000 × g to pellet the stromal vascular fraction. The pellet was washed once with sterile PBS and centrifuged at 1000 × g. The pellet was resuspended in 1 ml of AdMSC media (low glucose DMEM containing L-glutamine and supplemented with essential and non-essential amino acids (Gibco), 15% heat-inactivated fetal bovine serum (FBS) (Peak Serum), and penicillin/streptomycin/neomycin (PSN) (Sigma)). Resuspension was filtered through a 40 µm cell strainer (Fisher) to remove any non-dissociated tissue. Cells were plated in 10 cm dishes and grown in AdMSC media. 72 h later, cells were passaged at a 1:3 ratio and again every 3–4 days. For all experimentation, cells were used at passage 2 or 3.

### Flow cytometry

AdMSCs at passage 3 were washed three times with sterile PBS. Cells were incubated with 3 ml of Cellstripper (Corning) for up to 15 min, tapping on plate every 5 min to dislodge cells. Cells were scraped and pooled, then centrifuged for 5 min at 1000×*g* at 4C. Cells were resuspended in FACS buffer (1% hiFBS and 1 mM EDTA in sterile PBS) and centrifuged at 1000×*g* for an additional 3 min. The cell pellet was resuspended in FACS buffer and aliquoted into 1.5 ml tubes. The cells were incubated for 30 min on ice with following antibodies: Mouse IgG FITC (1:2000, Bio-Rad), CD34-FITC (1:1000, Bio-Rad), CD44-FITC (1:1000, Bio-Rad), CD45-FITC (1:1000, Bio-Rad), CD73-FITC (1:2000, BioLegend), CD90-FITC (1:100, Abcam), CD105 (1:100, Bio-Rad, with anti-rat 488 secondary, 1:500). Cells were centrifuged at 500×*g* for 30 s and washed three times with FACS buffer. On the final wash, cells were transferred into library tubes with 500 µl FACS buffer and placed on ice. Live/dead stain (Sytox AADvance, Invitrogen) was added at 1:100 dilution to all samples excluding reference controls. 10,000 live cells per sample were analyzed using the Cytek™ 4-laser Aurora Cytometer and data was processed in FlowJo, gating on single cells, live cells, then FITC-positive and FITC-negative cells.

### AdMSC stimulation

AdMSCs at passage 3 were plated at 100,000 cells/well in 6-well plates. The following day, cells were stimulated with either cytokines or brain homogenate. Cytokine-treated cells received media containing 10 ng/ml TNFα or 200 ng/ml IFNγ (R&D Systems) for 1, 4, 8, 12 or 24 h, followed by RNA isolation. Cells incubated in media containing 0.1% normal or 22L brain homogenate were treated for the same time course before RNA isolation. Control cells received media only.

### AdMSC migration assay

AdMSCs at passage 2 were stimulated for 24 h with media containing 10 ng/ml TNFα (R&D Systems). Cells were washed and incubated in serum-free media for 4 h. 1% RML or NBH were plated in serum-free media in a 24-well plate. 24-well inserts with a pore size of 8 µm (Greiner Bio-One) containing 25,000 serum-starved AdMSCs per insert were added to corresponding wells. Cells were incubated for 24 h at 37C, washed, then a cotton swab was used to gently remove cells from the top chamber of the insert. Cells were incubated for 1 h in crystal violet solution (0.2% crystal violet, 11% formaldehyde, 2% ethanol, 2% paraformaldehyde in H2O) and washed thoroughly with PBS. Four random areas were selected from each insert and cells were imaged and counted with a 10 × objective on an inverted microscope (Laxco) using SeBaView software.

### Isolation and prion infection of mixed glia

Zero to two-day old C57Bl/6 pups were euthanized and brains were extracted. Cortex was separated and cerebellum, meninges and midbrain were removed and discarded, and the brains were placed in MEM/EBSS containing 2 × PSN on ice. Cortical tissue was used for mixed glial cultures^38^. Media was removed and replaced with prewarmed dissociation media (MEM/EBSS, 2 × PSN and 1.5U/ml Dispase (Gibco)) and triturated with a Sigmacote (Sigma) coated glass pipet. The mixture was transferred to a beaker and stirred gently for 10 min. Tissue was allowed to settle and supernatant was removed and transferred to a tube on ice. DNase-I (4000 U/ml, Roche) was added to dissociation media and tissue was resuspended and stirred for an additional 10 min. Extractions were repeated by adding fresh dissociation media (without DNase-I) 2 to 4 additional times, depending on the amount of tissue, until only fibrous tissue remained in the bottom of the beaker. The cell supernatant was centrifuged for 10 min at 1000×*g* at 4C, media was aspirated from cell pellet and replaced with glial growth medium (MEM/EBSS, 10% FBS and 1% PSN). 10^6^ mixed glial cells were plate in 10 cm dishes. 24 h later, media was replaced. Media was changed weekly. For in vitro prion infection, mixed glia were plated at 100,000 cells per well in 6-well plates and infected with 0.1% normal or 22L brain homogenate once cells were 80–90% confluent. Media was removed 72 h later and cells were washed twice with PBS prior to fresh media being added to remove any residual brain homogenate. Media was changed weekly on infected cells. Cells were lysed after being infected for 7, 14, 21 and 28 days for western blot analysis.

### Immunofluorescence to characterize AdMSCs

Cells were fixed with cold 4% paraformaldehyde for 10 min and permeabilized with 0.1% Triton-X for 5 min, and incubated in PBS containing 10% normal goat or donkey serum for 1 h at room temperature (RT). Antibodies in 5% serum were incubated at 4C overnight. AdMSC markers include Oct3/4 (1:100, Abcam) and Vimentin (1:100, Sigma Aldrich). Following the overnight incubation, cells were washed and incubated with 555-conjugated anti-rabbit or anti-mouse secondary antibodies (1:500, Southern Biotech) and incubated in the dark at RT for 1 h. Slides were incubated in Hoechst stain (ThermoFisher, 1:2000 dilution) for three minutes, then mounted with ProLong Gold Antifade media (ThermoFisher) and cover slipped (Globe Scientific, #1).

### Co-culture of prion-treated mixed glia and BV2 cells with AdMSCs

For RNA assays, mixed glia were plated in glial growth medium (MEM/EBSS, 10% FBS and PSN) on 6-well dishes at 100,000 cells per well. Once confluent, cells were treated with media containing 0.1% NBH or 22L brain homogenate. 72 h later, media was removed, cells were washed twice with PBS, and fresh media was added. Four days later, media was changed to AdMSC media (low glucose DMEM containing L-glutamine and supplemented with essential and non-essential amino acids, 15% heat-inactivated FBS, and PSN). 6-well inserts with a pore size of 0.4 µm (Greiner Bio-One) containing 100,000 AdMSCs per insert were added to corresponding wells. Cells were incubated for an additional 7 days with or without AdMSC co-cultures before inserts were removed and glial RNA was isolated from all samples.

BV2 microglia were plated at 50,000 cells per well and exposed 24 h later to 0.1% NBH or RML. Three days later, media containing brain homogenates were removed and BV2 cells were washed twice with PBS and fresh media added. AdMSCs at passage 1 were stimulated 24-h prior to co-culturing by treatment with media containing 10 ng/ml TNFα (R&D Systems) for 24 h. After 24 h, media containing TNFα was removed, AdMSCs were washed three times with PBS, and stimulated AdMSCs were trypsinized and added to inserts above the BV2 cells at 6 days post-prion exposure. BV2 cells were co-cultured with or without AdMSCs for 24 h before RNA was isolated from the BV2 cells.

### Reverse transcriptase quantitative PCR analysis

RNA was extracted from cell culture 6-well dishes using cell scraping, QIAshredder and RNeasy extraction kits, in accordance with manufacturer’s protocol, including a DNase digestion step with the RNase free DNase kit (Qiagen, Valencia, CA). Purity and concentration were determined using a ND-1000 spectrophotometer (NanoDrop Technologies, Wilmington, DE). Following isolation and purification, 25 ng of RNA was reverse transcribed using the iScript Reverse Transcriptase kit (BioRad, Hercules CA). The cDNA was amplified within 24 h of reverse transcription using iQ SYBR Green Supermix (BioRad, Hercules CA). The corresponding validated primer sequences were used for each gene at 10 µM. The expression data was analyzed using the 2^−ΔΔCT^ method and normalized to expression of reference genes *β-actin* or *GAPDH*^68^. The fold difference was compared to control (normal brain homogenate treated) samples Validated primer sequences are as follows:

(TSG-6) 5′-GCTACAACCCACATGCAAAGGA-3′ (forward), 5′-CCGTACTTGAGCCGAATGTGC-3′ (reverse); (TGFβ1) 5′-CTTCAATACGTCAGACATTCGGG-3′ (forward), 5′-GTAACGCCAGGAATTGTTGCT-3′ (reverse); (IL1β) 5′-GCAGCAGCACATCAACAAG-3′ (forward), 5′-CACGGGAAAGACACAGGTAG-3′ (reverse); (TNFα) 5′-CCGATGGGTTGTACCTTGTC-3′ (forward), 5′-AGATAGCAAATCGGCTGACG-3′ (reverse); (CCL2) 5′-TTAAAAACCTGGATCGGAACCAA-3′ (forward), 5′-GCATTAGCTTCAGATTTACGGGT-3′ (reverse); (CCL5) 5′-GCTGCTTTGCCTACCTCTCC-3′ (forward), 5′-TCGAGTGACAAACACGACTGC-3′ (reverse); (C3) 5′-GAGCGAAGAGACCATCGTACT-3′ (forward), 5′-TCTTTAGGAAGTCTTGCACAGTG-3′ (reverse); (S100β) 5′-CGAGAGGGTGACAAGCACAAG-3′ (forward), 5′-CTTCCTGCTCCTTGATTTCCTCCA-3′ (reverse); (TMEM119) 5′-TCACCCAGAGCTGGTTCCATA-3' (forward), 5′-GAGTGACACAGAGTAGGCCA-3' (reverse); (Arg-1) 5′-CGTAGACCCTGGGGAACACTAT-3′ (forward), 5′-^TCCATCACCTTGCCAATCCC^-3′ (reverse); (IL-6) 5′-CTGCAAGAGACTTCCATCCAG-3′ (forward), 5 ′-AGTGGTATAGACAGGTCTGTTGG-3′ (reverse); (CD16) 5′-TTTGGACACCCAGATGTTTCAG-3′ (forward), 5′-GTCTTCCTTGAGCACCTGGATC-3′ (reverse); (NLRP3) 5′-CCTGGGGGACTTTGGAATCA-3′ (forward), 5′-GACAACACGCGGATGTGAGA-3′ (reverse); (β-actin) 5′-GCTGTGCTATGTTGCTCTAG-3′ (forward), 5′-CGCTCGTTGCCAATAGTG-3′ (reverse); (GAPDH) 5′-AGGAGAGTGTTTCCTCGTCC-3′ (forward), 5′-CCGTTGAATTTGCCGTGAGT-3′ (reverse). All RT-PCR was done following MIQE guidelines.

### Immunoblotting

Cell lysates were isolated using the protein lysis buffer (50 mM Tris, 150 mM NaCl, 2 mM EDTA, 1 mM MgCl2, 100 mM NaF, 10% glycerol, 1% Triton X-100, 1% Na deoxycholate, 0.1% SDS and 125 mM sucrose) supplemented with Phos-STOP and protease inhibitors (Roche). A BCA Protein Assay kit (Thermo Scientific) was used to quantify protein concentration of lysates, and 250 µg or 500 µg protein was digested with 20 µg/ml proteinase K (PK) (Roche) for PrP^Sc^ blots for 1 h at 37C. For brain homogenate control, 2 µl of a 20% brain homogenate (diluted in PBS) was treated with PK. Digestion was terminated with 2 mM PMSF and lysates were spun at 40,000×*g* for 1 h at 4C before being loaded on a gel. For PrP^C^ blots, 20 µg of samples were used. Samples were run using 4–20% acrylamide SDS page gels (BioRad) and then transferred onto PVDF blotting paper (MilliPore). Primary antibody Bar-224 (Cayman Chemical Company) was used at 1:1,000 dilution for PrP^Sc^ blots and 1:5000 dilution for PrP^C^ blots. HRP-conjugated secondary antibodies were used at a concentration of 1:5000 (Vector Laboratories). For PrP^C^ blots, loading control GAPDH was ran at a 1:5000 dilution (MilliPore), with HPR-conjugated secondary at 1:5000 dilution (Southern Biotech). The protein antibody complex was visualized using SuperSignal West Pico PLUS Chemiluminescent Substrate (Thermo Scientific) and visualized with the BioRad ChemiDoc MP.

### Scrapie cell assay

Protocol adapted from Bian et al. 2010. Primary glial cells at passage 1 were plated in 24-well plates at 25,000 cells/well and infected with 0.1% 22L or normal brain homogenate for 72 h, as described above. 7 days post-infection they were co-cultured with 25,000 AdMSCs on passage 3, as described above. 7 days later, AdMSC inserts were removed and plates were trypsinized and 20,000 cells were transferred to each well of a 96-well ELISpot plate (Millipore). Liquid was removed from the plates via a bottom vacuum and plates were thoroughly dried at 50 °C. Plates were treated with cell lysis buffer (50 mM Tris–HCl, 150 mM NaCl, 0.5% IGEPAL-CA630, 0.5% Sodium Deoxycholate in H2O pH = 7.6) containing 5ug/ml PK (Sigma Aldrich) and incubated for 90 min at 37 °C on a shaker. The digestion was terminated with 2 mM phenylmethylsulphonyl fluoride (PMSF, Thermo Fisher) at room temperature on a shaker for 20 min. The vacuum was applied and plates were incubated with 3 M Guanidinium Thiocyanate (Research Products International) in 10 mM Tris–HCl (Sigma) (pH 8.0) for exactly 10 min at room temperature on a shaker. Digestion was terminated with vacuum and application of 150 µl PBS to each well. Plates were washed thoroughly, then blocked for 1 h at room temperature with 5% Superblock (Pierce, Rockford, IL) on a shaker. The vacuum was applied and plates were incubated overnight at 4C with primary antibody Sha31 (Cayman Chemical Company), diluted 1:5000 in TBST (Tris-Buffered Saline with Triton-X). Plates were washed with TBST and incubated for 1 h at room temperature with secondary antibody, AP-α-Mouse IgG (Southern Biotechnology Associates, Birmingham, AL), diluted 1: 5000 in TBST. Plates were washed thoroughly and dried overnight. NBT/BCIP tablet (Roche) in ultrapure water was added to plates and incubated in the dark for 30 min. Plates were washed and dried overnight at 4C. Plates were scanned with a ImmunoSpot S6-V analyzer (Cellular Technology Ltd, Shaker Heights, OH), and determined spot numbers using ImmunoSpot5 software (Cellular Technology Ltd, Shaker Heights, OH).

## Supplementary Information

Supplementary Information.

## Data Availability

The data that support the current study are available from the corresponding author upon request.
