# Supplementary material for: Adipose-derived mesenchymal stromal cells decrease prion-induced glial inflammation in vitro
Source: Sci Rep. 2022 Dec 29;12:22567. doi: 10.1038/s41598-022-26628-7 (PMC9800558; doi:10.1038/s41598-022-26628-7)
Supplement: Supplementary file 1 — Supplementary Information. [file 41598_2022_26628_MOESM1_ESM.docx]

**Supplemental Data**

**Enzyme-Linked Immunosorbent Assay**

AdMSCs at passage 3 were treated with media containing 10 ng/ml TNFα for 24 hours. Media was then removed and cells were washed three times with PBS to remove any residual TNFα. 24 hours later, media was removed and an ELISA was used to identify TNFα in the media. ELISAs were performed as per manufacturer’s instructions (R&D Biosystems).


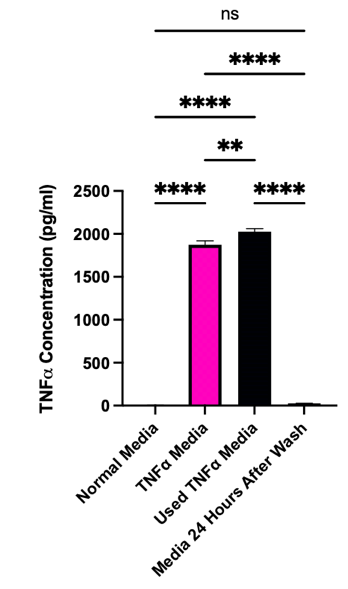


**Figure S1.** TNFα is significantly detectable by ELISA in media from AdMSCs that have been stimulated for 24 hours with TNFα, but not after thorough washing and media change. One-Way ANOVA and Tukey test, p<0.05, error bars = SEM. * p< 0.05, ** p< 0.01, *** p< 0.001, **** p< 0.0001, ns = not significant.


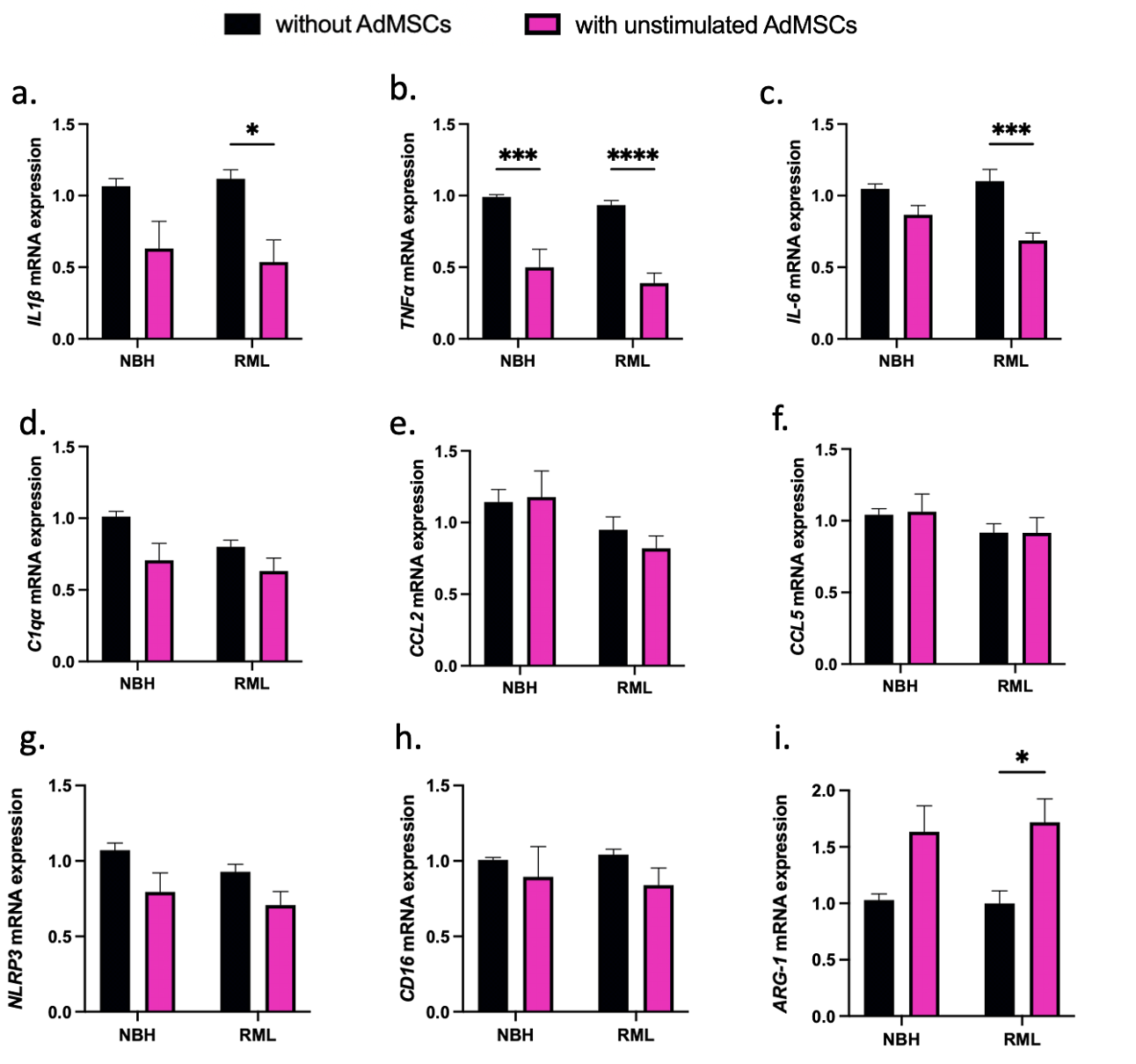


**Figure S2.** BV2 microglia were infected with 0.1% RML brain homogenate or normal brain homogenate (NBH) for 6 days, then incubated for 24 hours with unstimulated AdMSCs. This resulted in a decrease in **(a)** IL1β, **(b)** TNFα and **(c)** IL-6 mRNA, but not **(d)** C1qα, **(e)** CCL2, **(f)** CCL5, **(g)** NLRP3, or **(h)** CD16. **(i)** An increase was seen in the M2 marker Arg-1. Three biological replicates, each with three technical replicates, all analyses normalized to *β-actin*. Two-way ANOVA and Tukey test, p<0.05, error bars = SEM. * < 0.05, ** < 0.01, *** < 0.001, **** < 0.0001


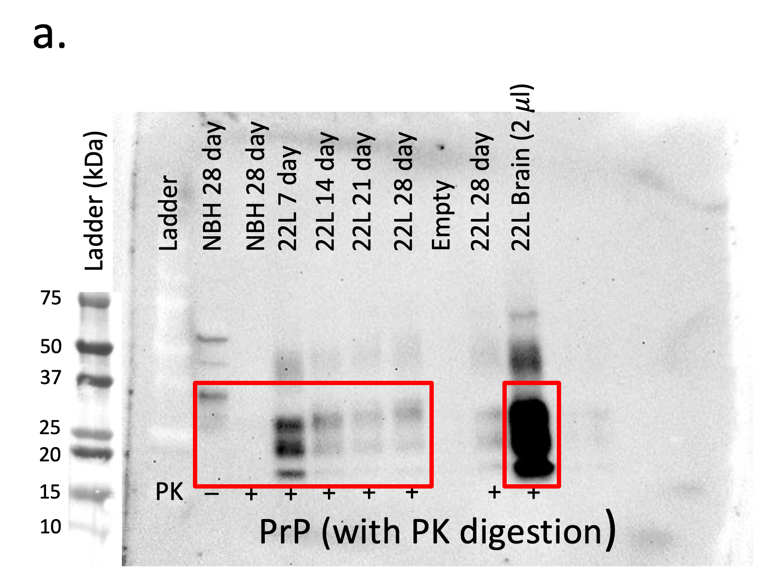


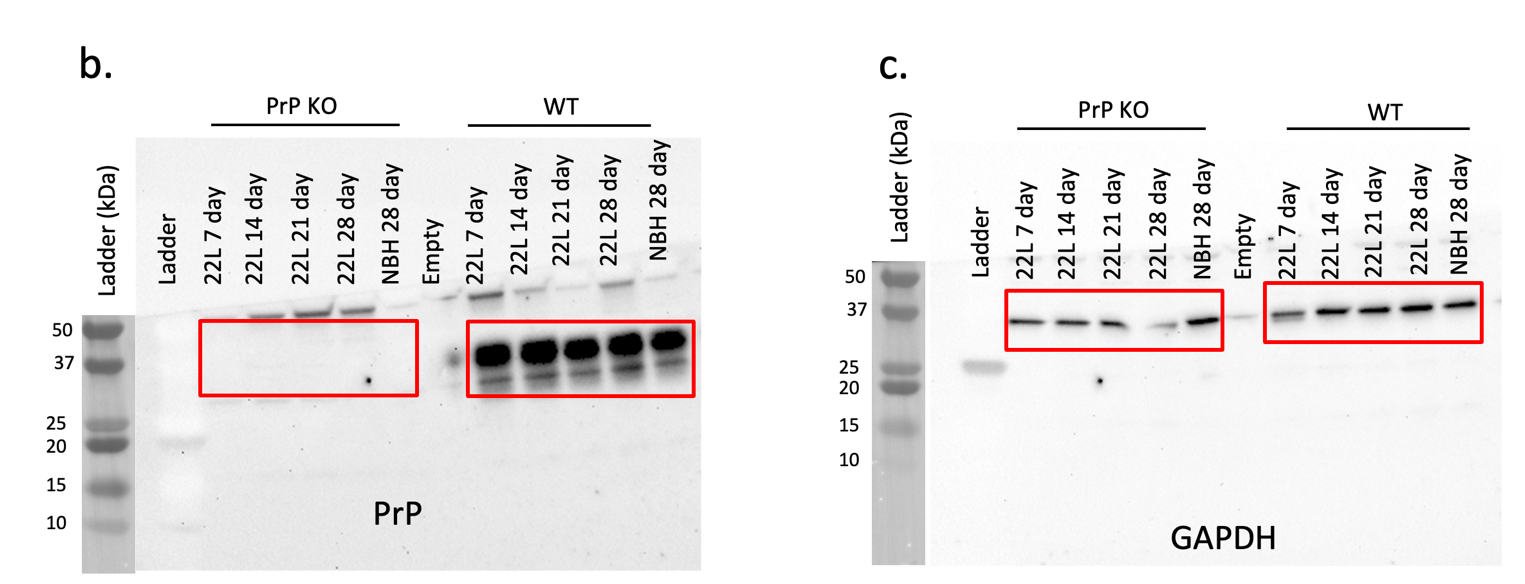

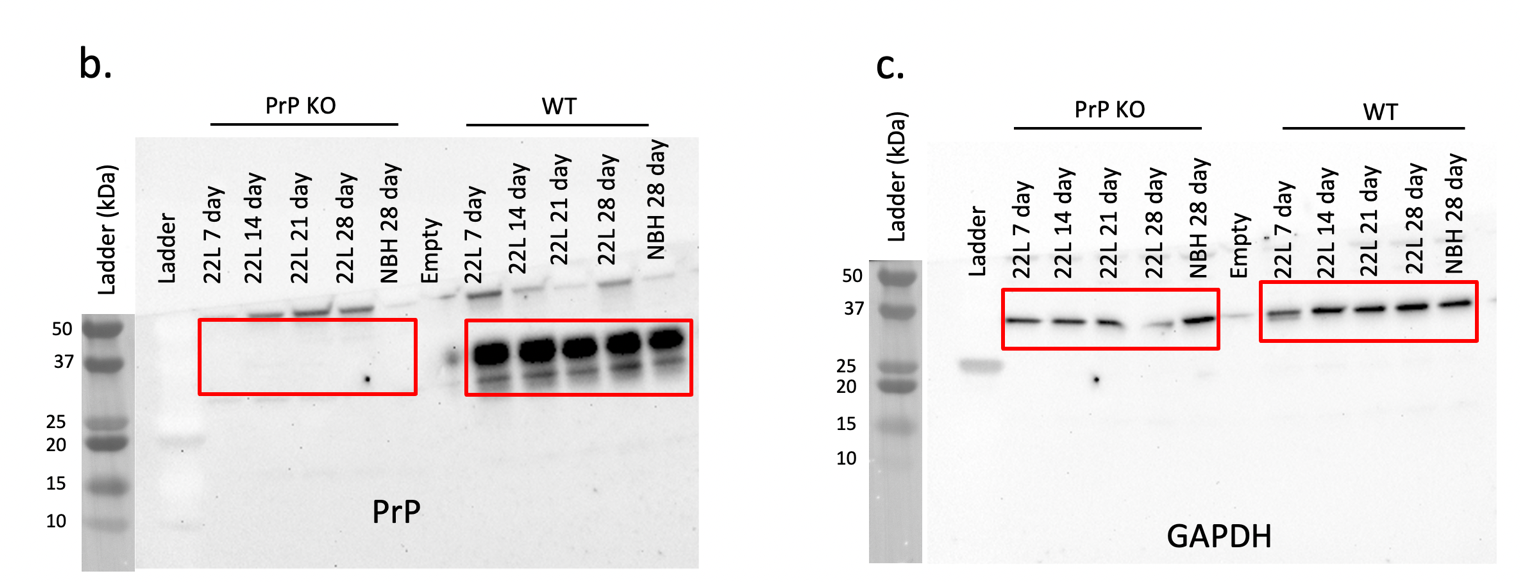


**Figure S3.** Full western blots for infected glial cultures, Figure 4. **(a)** PrP^Sc^ in infected mixed glia. **b)** PrP^C^ or **(c)** GAPDH in infected glia, no PK digestion. Sections of the blots that are used in Figure 4 are outlined with red boxes.


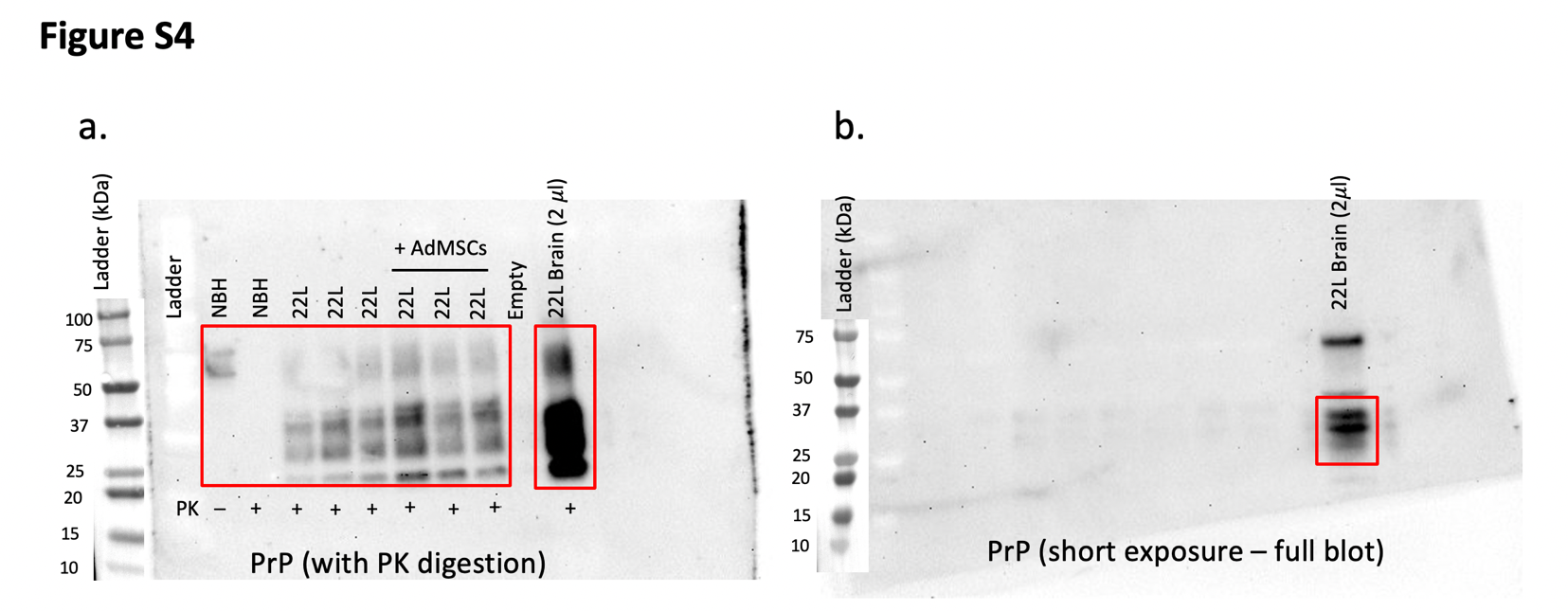


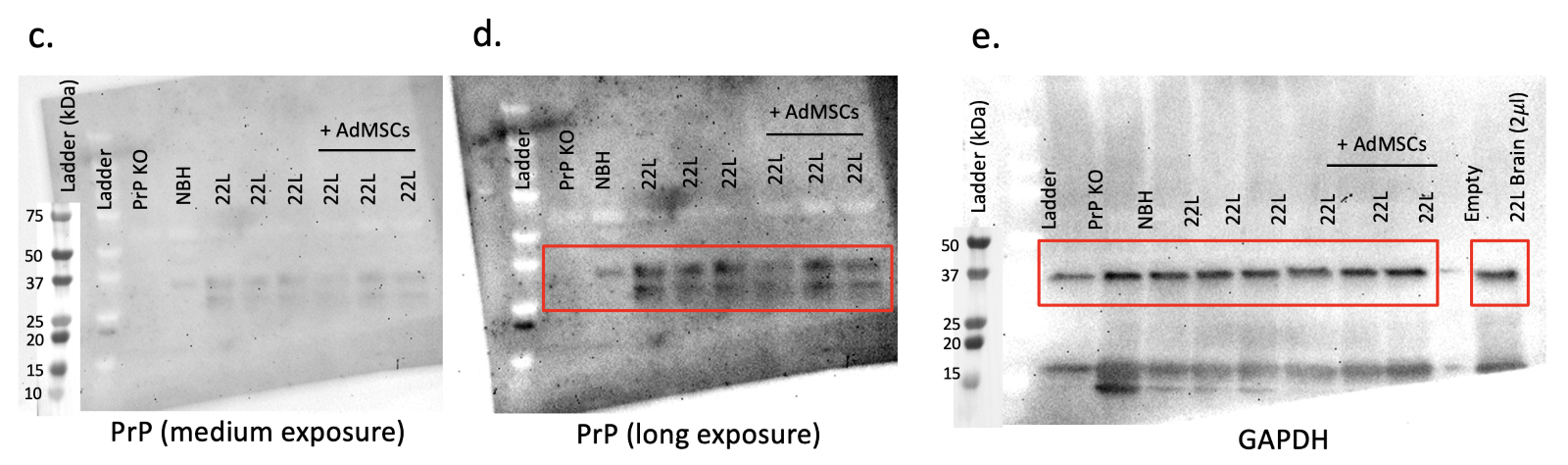


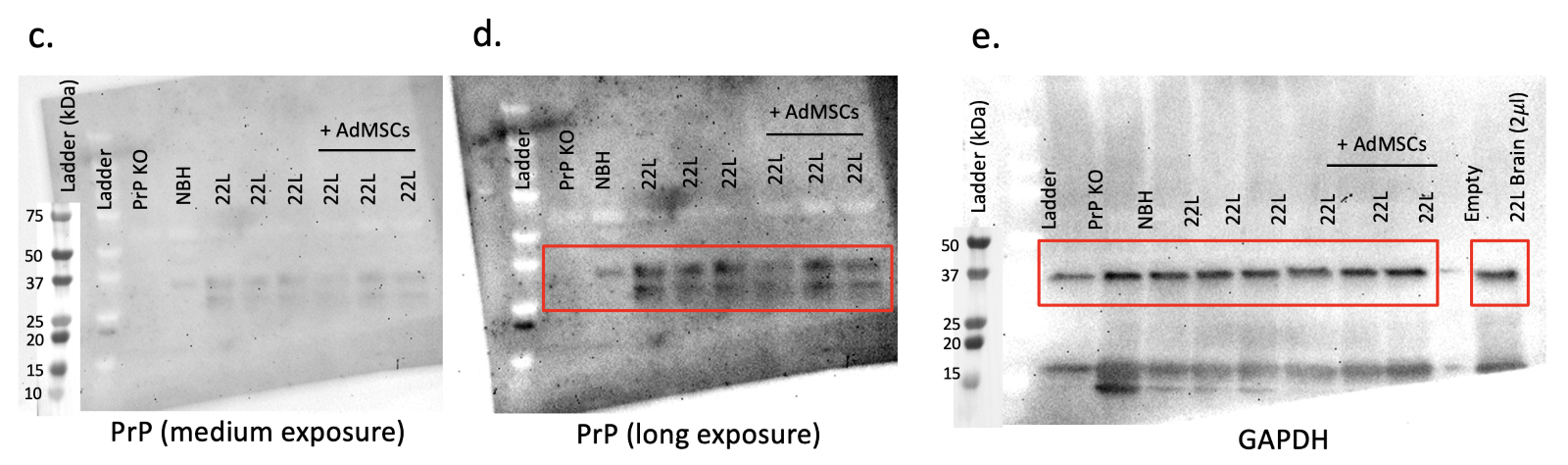


**Figure S4.** Full western blots for infected glial cultures, Figure 5. Infected glia with and without AdMSCs. (a) PrP^Sc^, (b) PrP^C^ short exposure with brain homogenate control, (c) PrP^C^ long exposure without brain homogenate control, (d) PrP^C^ long exposure adjusted contrast in ImageJ, (e) GAPDH. Sections of the blots that are used in Figure 4 are outlined with red boxes.
